# Adherence to Venous Thromboembolism Prophylaxis Guidelines in Medical Patients

**DOI:** 10.7759/cureus.105386

**Published:** 2026-03-17

**Authors:** Ning Lee, Min Ning Chu, Dasitha Narangoda

**Affiliations:** 1 Medicine, Letterkenny University Hospital, Letterkenny, IRL; 2 Internal Medicine, Letterkenny University Hospital, Letterkenny, IRL; 3 Psychiatry, Beaumont Hospital, Dublin, IRL

**Keywords:** padua prediction score, physician guideline adherence, safety patient, thromboprophylaxis, venous thromboembolism (vte)

## Abstract

Background

Venous thromboembolism (VTE) is a major cause of preventable morbidity and mortality among hospitalized medical patients. Despite the availability of validated risk assessment strategies and established prophylactic measures, variation in clinical practice persists, leading to both under- and over-utilisation of thromboprophylaxis.

Methods

A retrospective single-centre clinical audit was conducted over a two-month period at Letterkenny University Hospital, Ireland. Medical inpatients were reviewed 24 hours after admission. VTE risk was assessed using the Padua Prediction Score alongside bleeding risk evaluation. Prescribed pharmacological and mechanical thromboprophylaxis were compared with local hospital guidelines.

Results

A total of 100 patients were included (mean age 70.9 ± 19.1 years). Fifty-eight patients (58%) were classified as high VTE risk. Among high-risk patients, 37 (64%) received inappropriate prophylaxis. Among low-risk patients (n = 42), 24 (57.1%) were managed inappropriately, with unnecessary pharmacological prophylaxis being the most common reason for non-adherence. Contributing factors included undocumented weight, incorrect dose adjustment, and absence of documented VTE risk assessment.

Conclusions

Adherence to VTE prophylaxis guidelines was suboptimal, with both under- and over-prescription identified. Structured risk assessment tools and improved admission documentation may enhance compliance and patient safety.

## Introduction

Venous thromboembolism (VTE), comprising deep vein thrombosis and pulmonary embolism, remains a leading cause of preventable morbidity and mortality among hospitalised medical patients. Medical inpatients are at increased risk due to immobility, acute illness, advanced age, and multiple comorbidities, with a substantial proportion of VTE events occurring during or shortly after hospitalisation [1].

Strong evidence supports the use of thromboprophylaxis to reduce VTE incidence in at-risk medical patients without a disproportionate increase in bleeding complications. Accordingly, international and national guidelines, including those from the American College of Chest Physicians, the American Society of Hematology, National Institute for Health and Care Excellence (NICE), and the Irish Health Service Executive, recommend routine VTE risk assessment on admission and the use of risk-stratified pharmacological and/or mechanical prophylaxis [2]. The Padua Prediction Score is a validated tool widely used to identify hospitalised medical patients at high risk of VTE and to guide prophylaxis decisions [3].

Despite clear recommendations, adherence to VTE prophylaxis guidelines in routine clinical practice remains inconsistent. Large observational studies have demonstrated both under-prescription of thromboprophylaxis in high-risk patients and inappropriate over-prescription in low-risk patients, exposing individuals to avoidable thrombotic or bleeding complications [4].

In Ireland, VTE prevention is recognised as a national patient safety priority, with hospital policies mandating documented risk assessment and appropriate prophylaxis for adult inpatients [5]. However, contemporary data evaluating real-world adherence to these policies in medical inpatients are limited. The aim of this audit was to evaluate adherence to VTE prophylaxis guidelines in hospitalised medical patients. The objectives were to determine the proportion of patients receiving guideline-concordant prophylaxis, identify patterns of inappropriate prescribing, and assess documentation of VTE risk, bleeding risk, weight, and renal function as potential markers of adherence [6].

## Materials and methods

A retrospective single-centre clinical audit was conducted at Letterkenny University Hospital, Ireland, over a two-month period. Adult medical inpatients admitted were eligible for inclusion. Patients were reviewed 24 hours after admission to allow adequate time for initial risk assessment and prescribing decisions.

Patients were excluded if they were receiving therapeutic anticoagulation at admission, had a length of stay of less than 24 hours, were admitted primarily for acute stroke, or had underlying haematology or oncology diagnoses, in accordance with local hospital VTE policy.

Data were collected from electronic medical records and medication prescription charts. Variables recorded included patient demographics, documented VTE, and bleeding risk assessments, body weight, renal function, and presence of contraindications to pharmacological prophylaxis. Details of prescribed pharmacological thromboprophylaxis, including drug choice and dose, as well as prescribed and applied mechanical prophylaxis, were recorded.

VTE risk was assessed using the Padua Prediction Score (≥4 classified as high risk). No patients with COVID-19 infection were included in the study cohort, and the Padua score was applied retrospectively without modification [7]. All patients diagnosed with COVID-19 were classified as high risk for VTE in accordance with contemporaneous local hospital guidance. Bleeding risk was assessed based on documented contraindications to anticoagulation, including active bleeding, thrombocytopenia, or recent major bleeding events.

Appropriateness of thromboprophylaxis was determined by comparing the prescribed pharmacological and mechanical thromboprophylaxis with local hospital guidelines. Inappropriate pharmacological prophylaxis was further categorised as omission (no prophylaxis prescribed despite indication) or insufficient dosing, defined as administration of pharmacological thromboprophylaxis at a dose lower than the standard recommended prophylactic dose [8]. Appropriate management was defined as correct risk stratification and correct use of pharmacological and/or mechanical prophylaxis, including appropriate dose adjustment for body weight and renal function.

Data analysis was performed using descriptive statistics. Continuous variables were summarised as mean ± standard deviation or median with interquartile range, as appropriate. Categorical variables were expressed as frequencies and percentages. Statistical analysis was conducted using Microsoft Excel (Microsoft® Corp., Redmond, WA) and IBM SPSS Statistics version 29 (IBM Corp., Armonk, NY). This audit was conducted in accordance with institutional clinical audit standards and did not require formal ethical approval.

Risk assessment and thromboprophylaxis decisions were guided by the hospital VTE prevention protocol, which incorporates the Padua Prediction Score and bleeding risk assessment (Table 1). No proprietary or licensed risk assessment tools were used in this study.

**Table 1:** VTE prevention protocol for hospitalized medical patients incorporating the Padua Prediction Score Adapted from the VTE Prevention Protocol for In-Patients Aged 16 Years or Over developed by the Health Service Executive (HSE) [9]. VTE, venous thromboembolism

Step	Assessment	Criteria	Recommended Action
1	VTE Risk Assessment (Padua Prediction Score)	Padua score ≥ 4 or COVID-19 patient	Patient considered at risk for VTE → proceed to bleeding risk assessment
		Padua score ≤ 3	Low VTE risk → no pharmacological prophylaxis; encourage mobilization
2	Bleeding Risk Assessment	Contraindication to anticoagulation present	High bleeding risk → use mechanical prophylaxis (e.g., compression stockings or intermittent pneumatic compression)
3	Pharmacological Prophylaxis	Based on patient factors	Proceed to pharmacological prophylaxis
4	Prescribe Prophylaxis	Based on patient factors	Administer LMWH or heparin according to body weight, renal function, and clinical condition
5	Additional Preventive Measures	All hospitalized patients	Encourage early mobilization, maintain adequate hydration, and reassess VTE risk during admission

## Results

A total of 100 medical inpatients were included. The mean age was 70.9 ± 19.1 years, with a median age of 76.5 years. 58 patients (58%) were classified as high VTE risk, while 42 patients (42%) were classified as low VTE risk.

Among high-risk patients, 37 of 58 (64%) received inappropriate thromboprophylaxis. Reasons for inappropriate management included incorrect dose adjustment for weight (n = 18), pharmacological thromboprophylaxis prescribed at a lower-than-recommended prophylactic dose (“insufficient dosing”) (n = 12), failure to prescribe mechanical prophylaxis in patients with bleeding risk where pharmacological prophylaxis was contraindicated (n = 5), and incorrect dose adjustment in renal impairment (n = 2). Reasons for inappropriate thromboprophylaxis among high-risk patients are summarised in Table 2.

**Table 2:** Reasons for inappropriate thromboprophylaxis among high-risk patients High VTE risk was defined using the Padua Prediction Score [1]. Prophylaxis was assessed according to guideline recommendations and local policy [9]. VTE, venous thromboembolism

Number of Patients	Reason for Inappropriate Thromboprophylaxis
18	Incorrect dose for weight
12	Insufficient pharmacological prophylaxis
5	Failure to prescribe mechanical prophylaxis in patients with bleeding risk where pharmacological prophylaxis was contraindicated
2	Incorrect dose adjustment for renal function (in patient with renal impairment)

Among low-risk patients, 24 of 42 (57.1%) received inappropriate management. Over-prescription of pharmacological prophylaxis was the most common reason for non-adherence (n = 22). Two patients with a contraindication to anticoagulation due to bleeding risk received inappropriate pharmacological prophylaxis. Reasons for inappropriate thromboprophylaxis among low-risk patients are outlined in Table 3.

**Table 3:** Reasons for inappropriate thromboprophylaxis among low-risk patients Low VTE risk was defined using the Padua Prediction Score [1]. Prophylaxis was assessed according to guideline recommendations and local policy [9]. VTE, venous thromboembolism

Number of Patients	Reason for Inappropriate Thromboprophylaxis
22	Over-prophylaxis with low-molecular-weight heparin (patient is low risk for VTE, and enoxaparin was prescribed).
2	Risk of bleeding, enoxaparin prescribed but contraindicated (the patient was low risk for VTE as well).

## Discussion

This audit demonstrated suboptimal adherence to VTE thromboprophylaxis guidelines among hospitalised medical patients, with clinically significant levels of both under-prescription in high-risk individuals and over-prescription in low-risk patients. These findings are consistent with international studies reporting persistent gaps between guideline recommendations and real-world clinical practice [10].

Although the majority of high-risk patients received some form of thromboprophylaxis, more than one-third did not receive guideline-concordant care. Incorrect dose selection, particularly related to undocumented body weight and renal impairment, was the most frequent contributor to inappropriate prescribing. Similar deficiencies have been reported in large observational studies, highlighting the importance of accurate clinical documentation in ensuring safe and effective prophylaxis [11]. In this audit, cases categorized as “insufficient prophylaxis” represented administration of thromboprophylaxis at a lower-than-recommended prophylactic dose rather than complete omission of prophylaxis.

Inappropriate pharmacological thromboprophylaxis was frequently prescribed to patients classified as low risk for VTE. Over-prescription in this group exposes patients to unnecessary bleeding risk without proven benefit and represents a potentially avoidable patient safety issue. Previous studies have similarly demonstrated that clinicians may overestimate VTE risk in the absence of structured risk assessment tools, leading to excessive anticoagulant use [6,11]. In several patients with contraindications to pharmacological prophylaxis, mechanical thromboprophylaxis was not prescribed despite the absence of documented contraindications, suggesting a gap in guideline implementation rather than clinical contraindication.

Incomplete documentation of formal VTE risk assessment was a recurring finding in this audit. The absence of documented risk stratification was commonly observed in patients with both under- and over-prescribing, suggesting it may be a marker of suboptimal adherence. Integration of validated tools, such as the Padua Prediction Score, into admission workflows has been associated with improved compliance and reduced variability in thromboprophylaxis practices [12,13].

In response to these findings, targeted quality improvement measures were implemented, including the incorporation of structured VTE and bleeding risk assessment tools into the medical admission proforma and focused clinician education. A planned re-audit will evaluate the effectiveness of these interventions and determine whether improvements are sustained over time [14,15].

## Conclusions

This audit demonstrates that adherence to established VTE thromboprophylaxis guidelines among hospitalised medical patients remains suboptimal, with clinically relevant levels of both under-prescription in high-risk patients and over-prescription in low-risk individuals. While most high-risk patients received some form of prophylaxis, a significant proportion did not receive care fully aligned with guideline recommendations. Unnecessary pharmacological prophylaxis in low-risk patients highlights potential exposure to avoidable bleeding risk and reflects variability in clinical decision-making. These findings underscore the ongoing challenges in translating evidence-based recommendations into routine clinical practice.

Incomplete documentation of VTE risk assessment, body weight, renal function, and bleeding risk was frequently observed in patients receiving inappropriate prophylaxis. While this audit cannot establish causality, such documentation gaps may serve as a marker of broader challenges in guideline adherence. Improving compliance will likely require a multifaceted approach, including structured risk assessment tools such as the Padua Prediction Score integrated into admission workflows, accurate and comprehensive documentation, targeted clinician education, and ongoing audit with feedback. Strengthening these processes is essential to optimise patient safety, reduce preventable hospital-associated VTE, and minimise unnecessary bleeding risk. A re-audit following implementation of these interventions will be valuable to assess their impact on clinical practice.
